# Immuno-virologic outcomes and immuno-virologic discordance among adults alive and on anti-retroviral therapy at 12 months in Nigeria

**DOI:** 10.1186/1471-2334-13-113

**Published:** 2013-03-01

**Authors:** Chuka J Anude, Emeka Eze, Henry C Onyegbutulem, Man Charurat, Mary-Ann Etiebet, Samuel Ajayi, Patrick Dakum, Oluyemisi Akinwande, Chris Beyrer, Alash’le Abimiku, William Blattner

**Affiliations:** 1 Institute of Human Virology, University of Maryland School of Medicine, Baltimore, Maryland, USA; 2 Johns Hopkins University Bloomberg School of Public Health, Baltimore, Maryland, USA; 3 University of Benin Teaching Hospital, Benin City, Edo State, Nigeria; 4 Asokoro District Hospital, Abuja, Federal Capital Territory, Abuja, Nigeria; 5 University of Abuja Teaching Hospital, Abuja, Federal Capital Territory, Abuja, Nigeria; 6 Institute of Human Virology Nigeria, Abuja, Nigeria

**Keywords:** Immuno-virologic outcomes, Immuno-virologic discordance, Anemia, Treatment failure, Sub-Saharan Africa, Nigeria, PEPFAR, Anti-retroviral therapy, Viral load testing

## Abstract

**Background:**

Predictors of immuno-virologic outcomes and discordance and their associations with clinical, demographic, socio-economic and behavioral risk factors are not well described in Nigeria since HIV viral load testing is not routinely offered in public HIV treatment programs.

**Methods:**

The HACART study was a multi-center observational clinic-based cohort study of 2585 adults who started HAART between April 2008 and February 2009. A total of 628 patients were randomly selected at 12 months for immuno-virologic analyses.

**Results:**

Virologic suppression rate (<400 copies/ml) was 76.7%, immunologic recovery rate (CD4 change from baseline ≥50 cells/mm^3^) was 77.4% and immuno-virologic discordance rate was 33%. In multivariate logistic regression, virologic failure was associated with age <30 years (OR 1.79; 95% CI: 1.17-2.67, *p*=*0*.*03*), anemia (Hemoglobin < 10 g/dl) (OR 1.71; 95% CI: 1.22-2.61, *p*=*0*.*03*), poor adherence (OR 3.82; 95% CI: 2.17-5.97, *p*=*0*.*001*), and post-secondary education (OR 0.60; 95% CI: 0.30-0.86, *p*=*0*.*02*). Immunologic failure was associated with male gender (OR 1.46; 95% CI: 1.04-2.45, *p*=*0*.*04*), and age <30 years (OR 1.50; 95% CI: 1.11-2.39, *p*=*0*.*03*). Virologic failure with immunologic success (VL^-^/CD4^+^) was associated with anemia (OR 1.80; 95% CI: 1.13-2.88, *p*=*0*.*03*), poor adherence (OR 3.90; 95% CI: 1.92-8.24, *p*=*0*.*001*), and post-secondary education (OR 0.40; 95% CI: 0.22-0.68, *p*=*0*.*005*).

**Conclusions:**

Although favorable immuno-virologic outcomes could be achieved in this large ART program, immuno-virologic discordance was observed in a third of the patients. Focusing on intensified treatment preparation and adherence, young patients, males, persons with low educational status and most importantly baseline anemia assessment and management may help address predictors of poor immuno-virologic outcomes, and improve overall HIV program impact. Viral load testing in addition to the CD4 testing should be considered to identify, characterize and address negative immuno-virologic outcomes and discordance.

## Background

Every HIV treatment program is confronted with the triple challenge of addressing gaps in coverage (access), retention in care and improving treatment outcomes. With HIV treatment access now a reality in most of sub-Saharan Africa (SSA), attention and concerns have rightly shifted to issues of retention in care, negative treatment outcomes, HIV drug resistance and treatment failures [1-4]. Treatment failure is known to result in greater treatment complexity and cost and in worsening morbidity and mortality rates among treated persons [5,6]. In resource-constrained settings, the World Health Organization (WHO) currently does not recommend routine HIV viral load (VL) testing, in part due to the cost and complex infrastructure needed for reliable results [5,7] but proposes the use of clinical and CD4+ lymphocyte-based criteria to guide treatment decision.

By the time most patients are diagnosed with immunologic failure (using 6 monthly CD4 cell counts) or clinical failure (by clinical history / examination or presence of new opportunistic infections), multiple drug resistance mutations may have developed compromising and complicating future drug treatment options [8,9]. Furthermore, there are groups of patients where viral replication is suppressed appropriately but without immunologic recovery. On the other hand, there are patients with immunologic recovery but without sustained viral load suppression. Thus, immuno-virologic discordance occurs when viral load test results used to assess virologic failure do not correspond to expected CD4 cell count results used to assess immunologic failure, and this discordance is associated with poor clinical outcomes.

Previous immuno-virologic outcomes studies in Africa [10-12] have been done in programs that commonly use Stavudine (d4T)-containing or Zidovudine (AZT)-containing first line highly active antiretroviral therapy (HAART). Little is known about immuno-virologic outcomes in US-government funded African public programs that do not perform routine viral load testing, but predominantly uses Tenofovir (TDF), an ARV with a different potency, safety, and resistance profile as part of a first line drug regimen. We sought to identify predictors of immuno-virologic outcomes and discordance among adults on ART who were still alive at 12 months.

## Methods

### Ethics statement

The study was reviewed and approved from all the Institutional Review Boards (IRB) of all three index hospitals in Nigeria, the Nigerian National Health Research Ethics Committee (NHREC), and the University of Maryland School of Medicine, Baltimore, Maryland, USA. Written informed consent was obtained from all the study participants after counseling and introduction of the study.

The HIV AIDS Care and Anti-Retroviral Therapy (HACART) study was a large multi-center observational clinic-based cohort study that described the predictors of loss to follow-up, immuno-virologic outcomes, immuno-virologic discordance and sub-optimal drug adherence within the AIDS Care and Treatment in Nigeria (ACTION) project. Funded by the U.S. Centers for Disease Control and Prevention Global AIDS Program (CDC GAP), the ACTION project is a tripartite partnership between the Institute of Human Virology of the University Of Maryland School Of Medicine, the Institute of Human Virology Nigeria, and the Nigerian Federal and State Ministries of Health.

**Figure 1:**
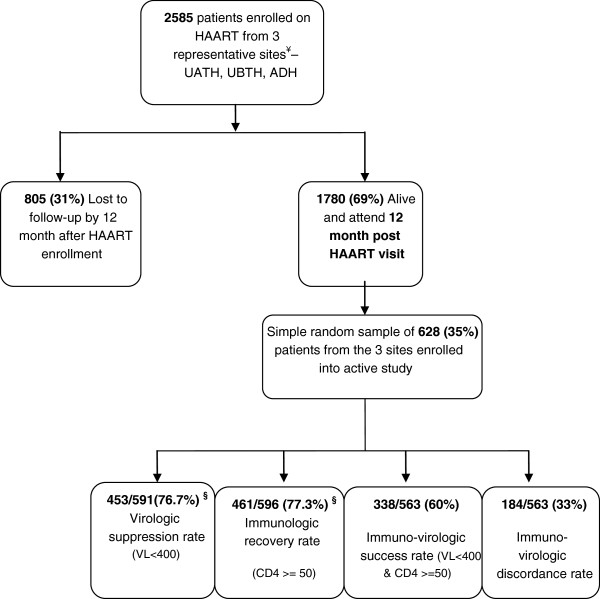
**Flow chart of Nigerian adults on ART recruited at 12 months and their 12**-**month outcomes.**

A cohort of 2585 initially ART naïve adults who started HAART between April 2008 and February 2009 were followed up for 12 months in three representative government hospitals in Nigeria: University of Abuja Teaching Hospital, Abuja (UATH), University of Benin Teaching Hospital, Benin (UBTH) and Asokoro District Hospital, Asokoro, Abuja (ADH). These sites consisted of a mix of tertiary (UATH and UBTH) and secondary (ADH) health facilities located in different regions of the country and serving different populations to increase generalizability. With 805(31%) lost to follow-up, a total of 628 out of the 1780 patients alive and active on the program at 12 months were randomly selected for in-depth interviews and laboratory work-up with detailed virologic and immunologic testing (Figure 1). There were no significant demographic and clinical differences between study patients at the 12 month time period and all the other patients alive and active at that 12 month time period (data not shown).

### Study design and patient management

Patients had their information recorded at baseline, were enrolled into ART care and followed up with prospective data abstracted from the medical records. Additional data not available at baseline was obtained at the 12-month visit. Blood work included HIV PCR RNA testing, CD4 cell count test, complete blood count and chemistry. Diagnosis of virologic and immunologic failure was made based on the results of the laboratory testing.

Before commencement of ART most patients underwent detailed history, physical examination, treatment preparation, HIV disclosure counseling, symptomatic screening for tuberculosis and laboratory work-up. Laboratory tests done at baseline included CD4 cell count, complete blood count, liver function tests and serum creatinine test. There were some missing laboratory results due to laboratory reagent shortage, or administrative errors. Cotrimoxazole was provided to eligible patients whose CD4 counts were less than 200 cells/ml. HAART was provided if patients satisfied an immunologic criteria (CD4 less than 200 cells/ml irrespective of WHO staging)^a^ or a clinical criteria (WHO stage 3 or 4 irrespective of CD4 cell count level). The choice of HAART combination was both guided by national treatment protocols and the discretion of the attending physician but generally consisted of one of three first line regimens (Tenofovir (TDF) + Lamivudine (3TC) + Nevirapine (NVP) / Efavirenz (EFV), Zidovudine (AZT) + 3TC + NVP / EFV, Stavudine (d4T) + 3TC + NVP / EFV. Patients who were anemic were preferably given TDF instead of AZT and persons who were co-infected with tuberculosis were also preferably given EFV instead of NVP. EFV was generally avoided in women of child-bearing age especially if they were not adherent to double contraception methods. A three months’ supply of drugs were provided (after an initial one month supply) with three monthly refill follow-ups and six monthly CD4 testing done together with adherence counseling sessions according to the national ART guidelines. Patients who default from scheduled clinic and pharmacy refill visits were followed-up by the home-based care team.

### Data collection

Standardized patient management and monitoring forms and interviews were used to capture detailed clinical, laboratory and demographic data, some of which were extracted from the CAREWare health information management system. Adherence was measured objectively by pharmacy refill records and subjectively by self-report. Residential distance from treatment sites to closest known town was determined in kilometers using the official Nigerian national road distance calculator and a unique online Nigerian distance tool at Globefeed.com (http://distancecalculator.globefeed.com/Nigeria_Distance_Calculator.asp). Since there were no validated data on socio-economic (SES) classifications in Nigeria, a quintile of class 1–5 was developed based on socio-economic scores calculated using 4 indicators commonly used in social epidemiology studies (highest educational achievement, employment status, household size, and monthly household income in *naira* (1 US$=150 Nigerian Naira).

### Outcome definition and ascertainment

Virologic failure (VL^-^) was defined as a HIV RNA level of equal or more than 400 copies / ml at the 12 month visit in line with local laboratory assay detection limits (viral load testing was done by quantitative polymerase chain reaction (PCR) for HIV-1 RNA in human plasma using Roche Amplicor version 1.5; Roche Diagnostics, Basal, Switzerland) and national treatment guidelines in Nigeria. Even though baseline HIV RNA levels were not done, it was expected that with adherence to potent HAART regimen, viral load levels would generally be undetectable (< 400 copies/ ml) by 12 months. Fresh samples from UBTH were processed and tested on site and fresh samples from AUTH and ADH were processed and tested at the National IHV reference laboratory onsite at ADH. Immunologic failure (CD4^-^) was defined with the WHO definition of “fall in CD4 count to baseline (pre-HAART) or below baseline levels (zero CD4 cell change)” or failure of CD4 cell to improve by at least 50 cells at 12 months [5,13]. A CD4 change of ≥ 50 cells from baseline was regarded as a sign of progressive immune recovery and has been used in other studies [14,15]. The CD4 cell count determination was done using Cyflow counter (Cyflow counter, Partec, GmbH, Gorlitz, Germany). Immuno-virologic failure is the combination of immunologic and virologic failure with virologic failure generally seen to precede immunologic failure. In this study, immuno-virologic discordance is said to occur when virologic failure is not followed by immunologic failure or when virologic success is not accompanied by immunologic success. Immuno-virologic discordance was divided into: virologic failure with immunologic success (VL^-^/CD4^+^) and immunologic failure with virologic success (VL^+^/CD4^-^).

### Statistical analysis

Predictors of interest (age, marital status, residential distance, baseline CD4 cell count, employment, tuberculosis history, regimen type, HIV disclosure, drug adherence and educational achievement) were categorized for data analysis. Hemoglobin was categorized either as a dichotomous variable using hemoglobin cut-off of 10 g/dl for anemia. Kruskal Wallis and Pearson χ^2^ tests were used to compare continuous and categorical variables respectively by site and outcome. Univariate and multivariate logistic regression was used to model the association between potential clinical, demographic, behavioral and socio-economic predictors on virologic and immunologic outcomes. To adjust for confounding, predictors were not included in the multivariable model if they yielded a univariate p-value of ≥ 0.2 according to the methods described by Maldonado and Greenland [16,17]. Covariates that were *a priori* known to be associated with the outcome of interest were forced into the model for face validity regardless of level of significance. Covariates in the final model were selected by step-wise backward selection procedure. Model goodness of fit was done by Hosmer-Lemeshow goodness of fit test.

All results were assessed at the 5% level of statistical significance for a 2-sided test. We also compared baseline demographic and laboratory parameters among patients that were sampled at 12 months and those who did not make it to 12 months. Some of the viral load and CD4 samples (6% and 5% respectively) were missing or were lost at random due to laboratory and / or administrative error. All missing data was assumed missing completely at random (MCAR) and multiple imputations (using sequential regression multivariate imputation (SRMI) method) using STATA did not show any differences in study result interpretation. All analyses were done with STATA version 11 (STATA Corp, College Station, Texas).

## Results

### Clinical, demographic, socioeconomic and behavioral descriptions of study population

Figure 1 shows the flow chart and Table 1 summarizes the baseline demographic, clinical, behavioral and socio-economic characteristics of the study participants. The median age at HAART initiation was 35 years (IQR: 30–41 years) and majority of the patients were females (n=400; 63.7%). The baseline median CD4 cell count was 142 cells/mm^3^ (IQR: 85–223), baseline median hemoglobin level was 10.9 g/dl (IQR: 9.3-12.4) and anemia based on hemoglobin less than 10 g/dl was common (n=192; 35.2%). Majority of the patients were married (n=359; 57.2%). HIV disclosure was high (n=595; 94.7%) and most of the patients (n=402; 64%) lived less than 50 kilometers from their respective treatment sites. More than half of the patients (n=321; 51.1%) were on a TDF-containing regimen at baseline with no substitutions within the 12 months. Even though low educational achievement levels with no formal education were seen in 301 (47.9%) patients, however, socio-economic indicators showed high employment levels (n=524; 83.4%).

**Table 1:** **Baseline**, **clinical and individual level characteristics of active adult study participants in Nigerian ART program **(**n **= **628**)

**Characteristics**					
	**Number of participants (% of total)**	**UATH (site %)**	**ADH (site %)**	**UBTH (site %)**	**p-value**^**§**^
**Gender**					0.31
Female	400 (63.7%)	128 (59.8%)	110 (67.1%)	162 (64.8%)	
Male	228 (36.3%)	86 (40.2%)	54 (32.9%)	88 (35.2%)	
**Age at HAART Initiation (years)**					
Median (Inter-quartile range)	35 (30 – 41)	34 (29 – 41)	34 (29 – 38)	36 (30 – 45)	0.001
< 30	190 (30.3%)	70 (32.7%)	55 (33.5%)	65 (26%)	
31 to 40	271 (43.1%)	89 (41.6%)	84 (51.2%)	98 (39.2%)	
41 to 50	120 (19.1%)	43 (20.1%)	20 (12.2%)	57 (22.8%)	
>51	47 (7.5%)	12 (5.6%)	5 (3.1%)	30 (12%)	
**Marital Status**					0.001
Single	146 (23.2%)	43 (20.1%)	47 (28.7%)	56 (22.4%)	
Married / Cohabiting	359 (57.2%)	136 (63.6%)	88 (53.7%)	135 (54%)	
Separated	45 (7.2%)	5 (2.3%)	9 (5.5%)	31 (12.4%)	
Divorced / Widowed	78 (12.4%)	30 (14%)	20 (12.2%)	28 (11.2%)	
**Currently married**					0.07
Yes	359 (57.2%)	136 (63.6%)	88 (53.7%)	135 (54%)	
No	269 (42.8%)	78 (36.4%)	76 (46.3%)	115 (46%)	
**Residential Distance (in kilometers)**					0.001
Median (inter-quartile range)	25 (10 – 80)	46.5 (20 – 90)	23 (15 – 30)	10 (5 – 130)	
< 50	402 (64%)	109 (50.9%)	133 (81.1%)	160 (64%)	
51 to100	80 (12.7%)	54 (25.2%)	16 (9.8%)	10 (4%)	
101 to 200	120 (19.1%)	44 (20.6%)	6 (3.7%)	70 (28%)	
>200	26 (4.1%)	7 (3.3%)	9 (5.5%)	10 (4%)	
**HIV disclosure**					0.34
Yes	595 (94.7%)	204 (95.3%)	158 (96.3%)	233 (93.2%)	
No	33 (5.3%)	10 (4.7%)	6 (3.7%)	17 (6.8%)	
**Tuberculosis History**					0.001
Yes	90 (14.3%)	18 (8.4%)	37 (22.6%)	35 (14%)	
No	538 (85.7%)	196 (91.6%)	127 (77.4%)	215 (86%)	
**Baseline CD4 cell count (n = 613)**					0.001
Median (inter-quartile range)	142 (85 – 223)	117 (72 – 175)	187 (111 – 266)	150 (88 – 241)	
< 50	88 (14.4%)	42 (19.8%)	15 (9.3%)	31 (13%)	
50 and < 200	336 (54.8%)	143 (67.5%)	74 (45.7%)	119 (49.8%)	
200 and <350	155 (25.3%)	19 (9%)	54 (33.3%)	82 (34.3%)	
350 and < 500	24 (3.9%)	4 (1.9%)	16 (9.9%)	4 (1.7%)	
>=500	10 (1.6%)	4 (1.9%)	3 (1.9%)	3 (1.3%)	
**Baseline Hemoglobin (g/dl) (n = 546)**					0.001
Median (inter-quartile range)	10.9 (9.3 – 12.4)	10.5 (8.8 – 11.9)	12.1 (10.8 – 13.9)	10.3 (8.9 – 11.6)	
> =10	354 (64.9%)	107 (60.1%)	131 (84%)	116 (54.7%)	
>=8 and <10 (Grade 1 anemia)	141 (25.8%)	57 (32%)	17 (10.9%)	67 (31.6%)	
>=7 and <8 (Grade 2 anemia)	33 (6.0%)	7 (3.9%)	7 (4.5%)	19 (9%)	
>=6.5 and <7 (Grade 3 anemia)	7 (1.3%)	3 (1.7%)	0 (0%)	4 (1.9%)	
< 6.5 (Grade 4 anemia)	11 (2.0%)	4 (2.3%)	1 (0.6%)	6 (2.8%)	
**Baseline anemia (Hemoglobin < 10 g/dl) (n = 546)**					
Yes	192 (35.2%)	71 (39.9%)	25 (16%)	96 (45.3%)	
No	354 (64.8%)	107 (60.1%)	131 (84%)	116 (54.7%)	
**Antiretroviral drug regimen at baseline**					0.001
ZDV / 3TC / NVP or EFV	257 (40.9%)	58 (27.1%)	76 (46.3%)	123 (49.2%)	
D4T / 3TC / NVP or EFV	50 (8.0%)	15 (7%)	10 (6.1%)	25 (10%)	
TDF / 3TC of FTC / NVP or EFV	321 (51.1%)	141 (65.9%)	78 (47.6%)	102 (40.8%)	
**Baseline Functionality**					0.001
Working (70-100%)		170 (81.3%)	71 (44.7%)	238 (96.8%)	
Ambulatory (50-70%)	479 (78%)	38 (18.2%)	78 (49.1%)	7 (2.8%)	
Bedridden (<50%)	123 (20%)	1 (%)	10 (6.3%)	1 (0.4%)	
	12 (2%)				
**Reason for ART eligibility**					
CD4 cell count < 200		188 (90.4%)	91 (56.5%)	159 (67.1%)	0.001
CD4 cell count < 350 and WHO Stage 3	438 (72.2%)	14 (6.7%)	55 (34.2%)	75 (31.7%)	
WHO Stage 4	144 (23.8%)	1 (0.5%)	14 (8.7%)	3 (1.3%)	
Other (PMTCT plus, etc.)	18 (3%)	5 (2.4%)	1 (0.6%)	0 (0%)	
	6 (1%)				
**Drug adherence by pharmacy refill record calculated over 12 months**					0.001
>= 95%	576 (91.7%)	202 (94.4%)	137 (83.5%)	237 (94.8%)	
< 95%	52 (8.3%)	12 (5.6%)	27 (16.5%)	13 (5.2%)	
**Drug adherence by self**-**report at 12 months**					0.08
>= 95%	544 (86.6%)	193 (90.2%)	135 (82.3%)	216 (86.4%)	
< 95%	84 (13.4%)	21 (9.8%)	29 (17.7%)	34 (13.6%)	
**Virologic suppression (<400 copies) (n = 591)**					0.001
Yes	453 (76.7%)	167 (78.4%)	94 (71.2%)	192 (78%)	
No	138 (23.3%)	46 (21.6%)	38 (28.8%)	54 (22%)	
**Immunologic recovery (CD4 cell change >=50) (n = 596)**					0.01
Yes	461 (77.4%)	178 (84.4%)	110 (74.3%)	173 (73%)	
No	135 (22.6%)	33 (15.6%)	38 (25.7%)	64 (27%)	
**Employment status**					0.001
Employed	524 (83.4%)	183 (85.5%)	116 (70.7%)	225 (90%)	
Unemployed	99 (15.8%)	27 (12.6%)	48 (29.3%)	24 (9.6%)	
Retired	1 (0.2%)	1 (0.5)	0 (0%)	0 (0%)	
Student	4 (0.6%)	3 (1.4%)	0 (0%)	1 (0.4%)	
**Highest educational achievement**					0.001
No formal education	301 (47.9%)	92 (43%)	80 (48.8%)	129 (51.6%)	
Primary education	136 (21.7%)	54 (25.2%)	22 (13.4%)	60 (24%)	
Secondary education	34 (5.4%)	14 (6.5%)	17 (10.4%)	3 (1.2%)	
Post-secondary / Tertiary / Advanced Education (M.Sc., Ph.D.)	157(25%)	54 (25.3%)	45 (27.4%)	58 (23.2%)	
**Socio**-**economic class**					0.001
Class 1	23 (3.7%)	13 (6.1%)	2 (1.2%)	8 (3.2%)	
Class 2	99 (15.6%)	27 (12.6%)	22 (13.4%)	50 (20%)	
Class 3	276 (44%)	79 (36.9%)	63 (38.4%)	134 (53.6%)	
Class 4	207 (33%)	88 (41.1%)	66 (40.3%)	53 (21.2%)	
Class 5	23 (3.7%)	7 (3.3%)	11 (6.7%)	5 (2%)	
**Current smoker**					0.65
Yes	11 (1.8%)	5 (2.3%)	3 (1.8%)	3 (1.2%)	
No	617 (98.2%)	209 (97.7%)	161 (98.2%)	247 (98.8%)	
**Current drug user**					0.24
Yes	1 (0.2%)	0 (0%)	1 (0.6%)	0 (0%)	
No	627 (99.8%)	214 (100%)	163 (99.4%)	250 (100%)	
**Current alcohol user**					0.001
Yes	53 (8.4%)	2 (0.9%)	29 (17.7%)	22 (8.8%	
No	575 (91.6%)	212 (99.1%)	135 (82.3%)	228 (91.2%)	
**Current herbal drug user**					0.03
Yes	20 (3.2%)	3 (1.4%)	10 (6.1%)	7 (2.8%)	
No	608 (96.8%)	211 (98.6%)	154 (93.9%)	243 (7.2%)	

*Categorical variables expressed as n* (%) *and continuous variables almost expressed as median *(*inter*-*quartile range*).

^§ ^p-values comparing study participant variables across the three study sites calculated from Kruskal-Wallis test statistic for continuous variables and Pearson χ^2^ test statistic for categorical variables.

There were differences in patients’ characteristics by site on all variables except marital status, HIV disclosure, adherence by self-report, smoking and drug use history. Males differed significantly from females in age at HAART initiation, marital status, TB history, baseline CD4 cell count, baseline hemoglobin count, employment status and smoking and alcohol history. This study was focused on outcomes among those alive at 12 months, and because of the random selection of patients, there were no major differences in the 628 patients selected for the study and the other 1152 who were alive at 12 months and not selected for the study (data not shown). There were differences in the baseline characteristics of age, residential distance from treatment site, baseline CD4 count, baseline anemia status and anti-retroviral drug regimen between the patients who were lost-to-follow-up and those alive and in the study at 12 months (Table 2). The outcomes and associations of patients lost-to-follow-up is in another related paper while this paper focuses on those who were alive at 12 months.

**Table 2:** **Comparison of baseline characteristics of Nigerian adults lost**-**to**-**follow**-**up and participating in the HACART study at 12 months**

**Characteristics**	**Lost-To-Follow-Up**	**Study participants**	**p-value**^**§**^
	**(n = 805)**	**(n = 628)**	
**Gender**			0.99
Female	513 (63.7%)	400 (63.7%)	
Male	292 (36.3%)	228 (36.3%)	
**Age at HAART Initiation (years)**			0.04
Median (Inter-quartile range)	33 (28–40)	35 (30–41)	
< 30	304 (37.7%)	190 (30.3%)	
31 to 40	306 (38%)	271 (43.1%)	
41 to 50	139 (17.3%)	120 (19.1%)	
>50	56 (7%)	47 (7.5%)	
**Marital Status (n = 2398)**^**‡**^			0.15
Single	184 (24.7%)	146 (23.2%)	
Married / Cohabiting	449 (60.4%)	359 (57.2%)	
Separated	40 (5.4%)	45 (7.2%)	
Divorced / Widowed	71 (9.5%)	78 (12.4%)	
**Currently married (n = 2398)**			0.23
Yes	449 (60.3%)	359 (57.2%)	
No	295 (39.7%)	269 (42.8%)	
**Residential Distance (in kilometers) (n = 2295) **^**‡**^			0.001
Median (inter-quartile range)	15 (10–75)	25 (10–80)	
< 50	486 (60.4%)	402 (64%)	
51 and < 100	134 (16.7%)	80 (12.7%)	
101 and <200	100 (12.4%)	120 (19.1%)	
>=200	13 (1.8%)	26 (4.1%)	
**HIV disclosure (n = 2082) **^**‡**^			0.28
Yes	481 (93.2%)	595 (94.7%)	
No	35 (6.8%)	33 (5.3%)	
**Tuberculosis History (n = 2025) **^**‡**^			0.76
Yes	72 (13.7%)	90 (14.3%)	
No	453 (86.3%)	538 (85.7%)	
**Baseline CD4 count (n = 2033) **^**‡**^			0.001
Median (inter-quartile range)	119 (54–218)	142 (85–223)	
< 50	137 (22.6%)	88 (14.4%)	
>=50 and < 200	301 (49.7%)	336 (54.8%)	
>=200 and <350	99 (16.3%)	155 (25.3%)	
>=350 and < 500	29 (4.8%)	24 (3.9%)	
>=500	40 (6.6%)	10 (1.6%)	
**Baseline Hemoglobin (g/dl) (n − 1606) **^**‡**^			0.001
Median (inter-quartile range)	9.9 (8–11.5)	10.9 (9.3-12.4)	
> =10	228 (48.6%)	354 (64.8%)	
>=8 and <10 (Grade 1 anemia)	127 (27.1%)	141 (25.8%)	
>=7 and <8 (Grade 2 anemia)	42 (9%)	33 (6%)	
>=6.5 and <7 (Grade 3 anemia)	14 (3%)	7 (1.3%)	
< 6.5 (Grade 4 anemia)	58 (12.4%)	11 (2%)	
**Baseline anemia (Hemoglobin < 10g/dl)**			0.001
Yes	241 (51.4%)	192 (35.2%)	
No	228 (48.6%)	354 (64.8%)	
**Antiretroviral drug regimen at baseline**			0.001
ZDV / 3TC / NVP or EFV	276 (34.3%)	275 (40.9%)	
D4T / 3TC / NVP or EFV	193 (24%)	50 (8%)	
TDF / 3TC of FTC / NVP or EFV	336 (41.7%)	321 (51.1%)	

*Categorical variables expressed as n *(%) *and continuous variables almost expressed as median *(*inter*-*quartile range*)

^§ ^p-values comparing study participant variables across the three study sites calculated from Kruskal-Wallis test statistic for continuous variables and Pearson χ^2 ^test statistic for categorical variables.

### Immuno-virologic parameters at 12 months on ART

Of the 591 with completed and validated viral load results, 453 had viral load results < 400 copies/ml giving a virologic suppression rate of 76.7% and thus a virologic failure rate of 23.4%. The median baseline CD4 cell count for patients with viral load < 400 copies / ml was 147 cells/mm^3^ (IQR: 87–225) compared to those with viral load > 400 at 122 cells /mm^3^ (IQR: 76–196) (p-value 0.04). Of 596 patients with CD4 cell counts (32 samples were not usable due to laboratory mishaps or administrative error) the immunologic failure rate was 22.7%. While 60% (n=338) had ideal immuno-virologic success (VL^+^/CD4^+^), immuno-virologic discordance rate was 33% (Table 2) with 17% (n=94) of the patients having had virologic failure with immunologic success (VL^-^/CD4^+^) and 16% (n= 90) of the patients having had immunologic failure with virologic success (VL^+^/CD4^-^).

### Predictors of virologic and immunologic outcomes at 12 months on ART

In the univariate logistic model (Table 3), a decreased odds of virologic failure was significantly associated with residential distance 51–100 kilometers (OR 0.45; 95% CI: 0.22-0.92) compared to residential distance less than 50 kilometers, and post-secondary/tertiary educational achievement (OR 0.53; 95% CI: 0.32-0.88) compared with no formal education. An increased odds of virologic failure was significantly associated with anemia with hemoglobin <10 g/dl (OR 1.59; 95% CI: 1.07-2.35) and with poor drug adherence of <95% both by pharmacy refill record (OR 3.76; 95% CI: 2.06-6.87) and self-reported adherence (OR 1.79; 95% CI: 1.07-2.99). After adjusting for site of study and other potential confounders, a decreased odds of virologic failure was still significantly associated with residential distance and post-secondary / tertiary educational achievement (OR 0.60; 95% CI: 0.30-0.86). Also, an increased odds of virologic failure remained significantly associated with age less than 30 years (OR 1.79; 95% CI: 1.17-2.67), anemia with hemoglobin less than 10 g/dl (OR 1.71; 95% CI 1.22-2.62) and adherence by pharmacy refill record < 95% (OR 3.82; 95% CI: 2.17-5.97).

**Table 3:** **Logistic regression analysis of predictors for virologic failure** (>**400 copies**/**ml**) **among Nigerian adult patients on ART**

**Characteristics**						
	**ALL**	**Virologic failure**	**Unadjusted**	**p**-**value**	**Adjusted**	**p-value**
	**(n = 591)**	**(n = 138)**	**OR (95% CI)**		**OR (95% CI)**	
**Gender**						
Female	374	87 (63%)	Ref		Ref	0.33
Male	217	51 (37%)	1.01 (0.68 – 1.5)	0.95	1.40 (0.72-2.07)	
**Age at HAART Initiation (years)**						
< 30	175	42 (30.4%)	1.51 (0.98-2.3)	0.06	1.79 (1.17-2.67)	0.03
>30	416	96 (69.6%)	Ref		Ref	
**Currently married**						
Yes	339	79 (43%)	0.99 (0.68-1.46)	0.98		
No	252	59 (57%)	Ref			
**Residential Distance (in kilometers)**						
< 50	372	90 (65%)	Ref		Ref	
51 to100	80	10 (7%)	0.45 (0.22-0.92)	0.03	0.44 (0.22-0.84)	0.04
>100	143	38 (28%)	1.17 (0.73-1.87)	0.49	1.37 (0.91-2.35)	0.24
**HIV disclosure**						
Yes	559	132 (96%)	0.75 (0.30-1.85)	0.53		
No	32	6 (4%)	Ref			
**Tuberculosis History**						
Yes	84	26 (19%)	1.58 (0.95-2.63)	0.08	1.20 (0.72-2.28)	0.46
No	507	112 (81%)	Ref		Ref	
**Baseline CD4 cell count (n = 613)**						
< 50	85	24 (17%)	Ref		Ref	
50 and < 200	317	79 (57%)	0.84 (0.49-1.44)	0.54	0.82 (0.47-1.46)	0.53
200 and <350	143	26 (19%)	0.56 (0.30-1.07)	0.08	0.54 (0.22-0.93)	0.08
350 and < 500	22	5 (4%)	0.75 (0.25-2.25)	0.61	0.48 (0.17-2.53)	0.24
>=500	24	4 (3%)	0.51 (0.16-1.64)	0.26	0.58 (0.19-2.6)	0.38
**Baseline anemia Hemoglobin <10 g/dl) (n = 591)**						
Yes	200	58 (42%)	1.59 (1.07-2.35)	0.02	1.71 (1.22-2.61)	0.03
No	391	84 (58%)	Ref		Ref	
**Antiretroviral drug regimen at baseline**						
ZDV / 3TC / NVP or EFV	247	56 (40.6%)	Ref		Ref	
D4T / 3TC / NVP or EFV	48	14 (10.1%)	1.32 (0.70-2.80)	0.34	1.35 (0.64-2.95)	0.45
TDF / 3TC of FTC / NVP or EFV	296	68 (50.3%)	0.94 (0.68-1.52)	0.93	0.90 (0.65-1.66)	0.79
**Baseline Functionality**						
Working (70-100%)	460	96 (71.1%)	Ref		Ref	
Ambulatory (50-70%)	106	34 (25.2% )	0.32 (0.09-1.06)	0.062	0.34 (0.12-1.18)	0.09
Bedridden (<50%)	11	5 (3.7%)	0.57 (0.16-1.99)	0.375	0.60 (0.14-1.87)	0.39
**Drug adherence by pharmacy refill record**						
>= 95%	543	114 (80%)	Ref		Ref	
< 95%	48	24 (20%)	3.76 (2.06-6.87)	0.001	3.82 (2.17-5.97)	0.001
**Employment status**						
Employed	496	111 (80%)	Ref		Ref	
Unemployed	90	26 (19%)	1.41 (0.85-2.33)	0.18	1. 37 (0.88-2.10)	0.26
Retired	1	0 (0%)	Omitted	Omitted	Omitted	
Student	4	1 (1%)	1.16 (0.12-11.2)	0.90	1.18 (0.15-10.6)	0.94
**Highest educational achievement**						
No formal education	283	80 (58%)	Ref		Ref	
Primary education	131	26 (19%)	0.63 (0.38-1.04)	0.07	0.66 (0.39-1.07)	0.06
Secondary education	30	7 (5%)	0.77 (0.32-1.87)	0.57	0.74 (0.31-1.79)	0.61
Post-secondary / Tertiary / Advanced education (M.Sc, PhD)	147	25 (18%)	0.53 (0.32-0.88)	0.01	0.60 (0.30-0.86)	0.02
**Socio**-**economic class**						
Class 1	23	6 (4%)	Ref			
Class 2	93	18 (13%)	0.68 (0.23-1.97)	0.48		
Class 3	260	55 (40%)	0.76 (0.29-2.02)	0.58		
Class 4	195	55 (40%)	1.11 (0.42-2.97)	0.83		
Class 5	20	4 (3%)	0.71 (0.17-2.98)	0.64		
**Current smoker**						
Yes	10	3 (2%)	1.42 (0.36-5.55)	0.62		
No	581	135 (98%)	Ref			
**Current drug user**						
Yes	1	0 (0%)	Omitted	Omitted		
No	590	138 (100%)	Ref			
**Current alcohol user**						
Yes	47	14 (10%)	1.44 (0.75-2.77)	0.28		
No	544	124 (90%)	Ref			
**Current herbal drug user**						
Yes	17	6 (4%)	1.83 (0.66-5.03)	0.24		
No	574	132 (96%)	Ref			

In the adjusted analysis, site was adjusted for in addition to the variables whose OR are included in the table.

A decreased odds of immunologic failure was not significantly associated with any predictor in the univariate or multivariate regression models (Table 4). However, an increased odds of immunologic failure was significantly associated with age less than 30 years (OR 1.55; 95% CI: 1.01-2.37) in the univariate model, and with male gender (OR 1.46; 95% CI: 1.04-2.45), age less than 30 years (OR 1.50; 95% CI: 1.11-2.39) in the multivariate regression model.

**Table 4:** **Logistic regression analysis of predictors for immunologic failure **(**Fall of CD4 count to or below baseline or CD4 cell count change **<**50 cells**/**mm**^**3 **^**at one year**) **among Nigerian adult patients on ART**

**Characteristics**						
	**ALL**	**Immunologic failure**	**Unadjusted**	**p**-**value**	**Adjusted**	**p-value**
	**(n = 596)**	**(n = 135)**	**OR (95% CI)**		**OR (95% CI)**	
**Gender**						
Female	377	84 (62%)	Ref		Ref	
Male	219	51 (38%)	1.06 (0.71-1.57)	0.78	1.46 (1.04-2.45)	**0**.**04**
**Age at HAART Initiation (years)**						
< 30	178	42 (31%)	1.55 (1.01-2.37)	0.04	1.50 (1.11-2.39)	**0**.**03**
>30	357	93 (69%)	Ref		Ref	
**Currently married**						
Yes	336	76 (56%)	0.99 (0.68-1.47)	0.98		
No	260	59 (44%)	Ref			
**Residential Distance (in kilometers)**						
< 50	376	92 (68%)	Ref		Ref	
51 to100	78	19 (14%)	0.99 (0.56-1.75)	0.98	1.19 (0.64-2.33)	**0**.**79**
>100	142	24 (18%)	0.63 (0.38-1.03)	0.07	0.68 (0.40-1.21)	**0**.**32**
**HIV disclosure**						
Yes	564	129 (22.9%)	0.78 (0.31-1.93)	0.59		
No	32	6 (18.8%)	Ref			
**Tuberculosis History**						
Yes	85	14 (29.6%)	0.64 (0.35-1.17)	0.14	0.66 (0.40-1.36)	**0**.**27**
No	511	121 (70.4%)	Ref		Ref	
**Baseline anemia (Hemoglobin <10 g/dl) (n = 546)**						
Yes	186	40 (20.4%)	1.26 (0.83-1.92)	0.27	1.05 (0.65-1.61)	**0**.**75**
No	410	95 (23.7%)	Ref		Ref	
**Antiretroviral drug regimen at baseline**						
ZDV / 3TC / NVP or EFV	242	58 (43%)	Ref			
D4T / 3TC / NVP or EFV	47	13 (9.6%)	1.21(0.59-2.45)	0.59	1.61 (0.69-3.53)	**0**.**29**
TDT / 3TC or FTC / NVP or EFV	307	64 (47.4%)	0.84 (0.56-1.25)	0.38	1.14 (0.77-1.53)	**0**.**83**
**Drug adherence by pharmacy refill record**						
>= 95%	549	122 (90.4%)	Ref		Ref	
< 95%	47	13 (9.6%)	1.34 (0.68-2.61)	0.39	1.42 (0.71-2.86)	**0**.**28**
**Employment status**						
Employed	496	110 (81.5%)	Ref			
Unemployed	95	25 (18.5%)	1.25 (0.76-2.07)	0.38		
Retired	1	0 (0%)	Omitted	Omitted		
Student	4	0 (0%)	Omitted	Omitted		
**Highest educational achievement**						
No formal education	288	64 (47.4%)	Ref			
Primary education	134	28 (20.7%)	0.92 (0.56-1.53)	0.76		
Secondary education	32	10 (7.4%)	1.59 (0.72-3.53)	0.25		
Post-secondary / Tertiary / Advanced education (M.Sc., Ph.D.)	142	33 (24.4%)	1.09 (0.67-1.76)	0.73		
**Socio**-**economic class**						
Class 1	22	4 (3%)	Ref			
Class 2	91	17 (12.6%)	1.03 (0.31-3.45)	0.96		
Class 3	262	68 (50.4%)	1.58 (0.52-4.82)	0.42		
Class 4	199	42 (31%)	1.20 (0.39-3.75)	0.75		
Class 5	22	4 (3%)	1.00 (0.22-4.63)	1.00		
**Current smoker**						
Yes	11	4 (97%)	1.98 (0.57-6.87)	0.28		
No	585	131 (3%)	Ref			
**Current drug user**						
Yes	0	0(0%)	Omitted	Omitted		
No	596	135 (100%)	Ref			
**Current alcohol user**						
Yes	50	8 (6%)	0.63 (0.29-1.37)	0.24		
No	546	127 (94%)	Ref			
**Current herbal drug user**						
Yes	17	6 (4%)	1.90 (0.69-5.24)	0.21		
No	579	129 (96%)	Ref			

### Predictors of Immuno-virologic discordance at 12 months on ART

In the multivariate regression model, an increased odds of VL^-^/CD4^+^ compared to VL^+^/CD4^+^ was significantly associated with poor adherence as monitored by pharmacy refill records (OR 3.90; 95% CI: 1.92-8.24) and anemia (Hemoglobin < 10 g/dl) (OR 1.80; 95% CI: 1.13-2.85) while decreased odds was associated with post-secondary educational achievement (OR 0.40; 95% CI: 0.22-0.68) (Table 5). Compared with VL^+^/CD4^+^, odds of VL^+^/CD4^-^ was significantly associated with male gender (OR 1.88; 95 CI: 1.05-3.11) and higher baseline CD4 > 200 (9.90: 95% CI: 2.79-35.10) (Table 6).

**Table 5:** **Logistic regression analysis of predictors of virologic discordance **(**VL** >**400 copies and CD4 change **>=**50 cell**) **compared to immuno**-**virologic success **(**VL **<**400 copies and CD4 change **>=**50 cells**) **at 12 month post**-**HAART among adult patients on ART in Nigeria**

**Characteristics**						
	**Immuno**-**virologic success (VL <400 copies and CD4 change >=50 cells)**	**Virologic discordance (VL >400 copies and CD4 change >=50 cells**)	**Unadjusted**	**p-value**	**Adjusted**	**p-value**
	**(n = 338)**	**(n = 94)**	**OR (95% CI)**		**OR (95% CI)**	
**Gender**						
Female	214 (63%)	58 (62%)	Ref		Ref	
Male	124 (37%)	36 (38%)	1.07 (0.67-1.72)	0.78	1.25 (0.72-2.01)	0.54
**Age at HAART Initiation (years)**						
< 30	269 (80%)	66 (70%)	1.65 (0.99-2.77)	0.06	1.58 (0.88-3.01)	0.08
>30	69 (20%)	28 (30%)	Ref		Ref	
**Currently married**						
Yes	188 (56%)	57 (61%)	1.23 (0.77-1.96)	0.39		
No	150 (44%)	37 (39%)	Ref			
**HIV disclosure**						
Yes	317 (93.8%)	89 (95%)	1.18 (0.43-3.22)	0.75		
No	21 (6.2%)	5 (5%)	Ref			
**Tuberculosis History**						
Yes	49 (14.5%)	17 (18.1%)	1.30 (0.71-2.39)	0.39	0.92 (0.47-1.86)	0.92
No	289 (85.5%)	77 (81.9%)	Ref		Ref	
**Baseline CD4 cell count (n = 613)**						
< 50	57 (16.9%)	19 (20.2%)	Ref		Ref	
50 and < 200	196 (58%)	59 (62.8%)	0.90 (0.50-1.64)	0.74	0.90 (0.56-1.61)	0.81
200 and <350	72 (21.3%)	13 (13.8%)	0.54 (0.25-1.19)	0.13	0.59 (0.20-1.38)	0.22
350 and < 500	9 (2.7%)	3 (3.2%)	1.00 (0.25-4.08)	1.0	0.58 (0.16-3.22)	0.65
>=500	4 (1.1%)	0 (0%)	Omitted			
**Baseline anemia (Hemoglobin <10 g/dl) (n = 546)**						
Yes	103 (30.5%)	40 (42.5%)	1.69 (1.05-2.70)	0.03	1.80 (1.13-2.85)	0.04
No	235 (69.5%)	54 (57.5%)	Ref		Ref	
**Antiretroviral drug regimen at baseline**						
ZDV / 3TC / NVP or EFV	139 (41%)	37 (39%)	Ref		Ref	
D4T / 3TC / NVP or EFV	24 (7%)	9 (10%)	1.41 (0.60-3.29)	0.43	1.21 (0.42-3.95)	0.59
TDF / 3TC of FTC / NVP or EFV	175 (52%)	48 (51%)	1.03 (0.64-1.67)	0.90	0.89 (0.46-1.43)	0.86
**Drug adherence by pharmacy refill record**						
>= 95%	322 (95%)	79 (84%)	Ref		Ref	
< 95%	16 (5%)	15 (16%)	3.82 (1.81-8.06)	0.001	3.90 (1.92-8.24)	0.001
**Employment status**						
Employed	288 (85.2%)	75 (79.8%)	Ref		Ref	
Unemployed	46 (13.6%)	18 (19.1%)	1.50 (0.82-2.74)	0.18	1.44 (0.77-2.56)	0.31
Retired	1 (0.3%)	0 (0%)	Omitted	Omitted	Omitted	
Student	3 (0.9%)	1 (1.1%)	1.28 (0.13-12.5)	0.83	1.33 (0.15-11.7)	0.87
**Highest educational achievement**						
No formal education	152 (45%)	58 (61.7%)	Ref		Ref	
Primary education	84 (24.9%)	18 (19.2%)	0.56 (0.31-1.02)	0.06	0.56 (0.29-1.01)	0.06
Secondary education	14 (4.1%)	5 (5.3%)	0.94 (0.32-2.72)	0.90	0.92 (0.27-2.49)	0.72
Post-secondary / Tertiary / Advanced education	88 (26%)	13 (13.8%)	0.40 (0.21-0.77)	0.006	0.40 (0.22-0.68)	0.004
**Socio**-**economic class**						
Class 1	14 (4.1%)	4 (4.3%)	Ref			
Class 2	54 (16%)	14 (14.9%)	0.91 (0.26-3.19)	0.88		
Class 3	147 (43.5%)	33 (35.1%)	0.79 (0.24-2.54)	0.69		
Class 4	110 (32.5%)	41 (43.6%)	1.30 (0.41-.4.19)	0.66		
Class 5	13 (3.9%)	2 (2.1%)	0.54 (0.08-3.45)	0.51		
**Current smoker**						
Yes	5 (1.5%)	1 (98.9%)	0.72 (0.08-6.21)	0.76		
No	333 (98.5%)	93 (1.1%)	Ref			
**Current alcohol user**						
Yes	28 (8.3%)	8 (8.5%)	1.03 (0.45-2.34)	0.94		
No	310 (91.7%)	86 (91.5%)	Ref			
**Current herbal drug user**						
Yes	7 (2.1%)	3 (3.2%)	1.56 (0.39-6.15)	0.53		
No	331 (97.9%)	91 (96.8%)	Ref			

**Table 6:** **Logistic regression analysis of predictors of immunologic discordance **(**VL** <**400 copies and CD4 change **<**50 cells**) **compared to immuno**-**virologic success **(**VL **<**400 copies and CD4 change **>=**50 cells**) **at 12 month post**-**HAART among adult patients on ART in Nigeria**

**Characteristics**						
	**Immuno**-**virologic success (VL <400 copies and CD4 change >=50 cells)**	**Immunologic discordance (VL <400 copies and CD4 change <50 cells)**	**Unadjusted**	**p-value**	**Adjusted**	**p-value**
	**(n = 338)**	**(n = 90)**	**OR (95% CI)**		**OR (95% CI)**	
**Gender**						
Female	214 (63%)	55 (61.1%)	Ref		Ref	
Male	124 (37%)	35 (38.9%)	1.09 (0.68-1.77)	0.90	1.88 (1.05-3.11)	0.04
**Age at HAART Initiation (years)**						
< 30	269 (80%)	27 (30%)	1.67 (0.99-2.82)	0.05	1.76 (0.91-3.20)	0.08
>30	69 (20%)	63 (70%)	Ref		Ref	
**Currently married**						
Yes	188 (56%)	54 (60%)	1.19 (0.75-1.92)	0.46		
No	150 (44%)	36 (40%)	Ref			
**HIV disclosure**						
Yes	317 (93.8%)	86 (95.6%)	1.42 (0.48-4.26)	0.53		
No	21 (6.2%)	4 (4.4%)	Ref			
**Tuberculosis History**						
Yes	49 (14.5%)	5 (5.6%)	0.35 (0.13-0.90)	0.03	0.43 (0.21-1.09)	0.08
No	289 (85.5%)	85 (94.4%)	Ref		Ref	
**Baseline CD4 cell count (n = 613)**						
< 50	57 (16.9%)	3 (3.3%)	Ref		Ref	
50 and < 200	196 (58%)	35 (38.9%)	3.39 (1.01-11.44)	0.049	3.17 (0.93-10.86)	0.07
200 and <350	72 (21.3%)	42 (46.7%)	11.08 (3.27-37.61)	0.001	9.90 (2.79-35.10)	>0.001
350 and < 500	9 (2.7%)	6 (6.7%)	12.67 (2.68-59.92	0.001	13.5 (2.59-70.13)	0.002
>=500	4 (1.1%)	4 (4.4%)	18.99 (3.12-115.86	0.001	19.1 (2.91-125.59)	0.002
**Baseline anemia (Hemoglobin <10 g/dl) (n = 546)**						
Yes	103 (30.5%)	24 (26.7%)	1.21 (0.72-2.03)	0.48	1.04 (0.55-1.88)	0.94
No	235 (69.5%)	66 (73.3%)	Ref		Ref	
**Antiretroviral drug regimen at baseline**						
ZDV / 3TC / NVP or EFV	139 (41%)	39 (43.3%)	Ref		Ref	
D4T / 3TC / NVP or EFV	24 (7%)	8 (8.9%)	1.18 (0.49-2.85)	0.70	1.74 (0.64-4.76)	0.25
TDF / 3TC of FTC / NVP or EFV	175 (52%)	43 (47.8%)	0.88 (0.54-1.43)	0.59	1.29 (0.72-2.22)	0.43
**Drug adherence by pharmacy refill record**						
>= 95%	322 (95%)	85 (94.4%)	Ref		Ref	
< 95%	16 (5%)	5 (5.6%)	1.18 (0.43-3.22)	0.75	1.20 (0.41-2.91)	0.64
**Employment status**						
Employed	288 (85.2%)	74 (82.2%)	Ref			
Unemployed	46 (13.6%)	16 (17.8%)	1.35 (0.73-2.53)	0.34		
Retired	1 (0.3%)	0 (0%)	Omitted			
Student	3 (0.9%)	0 (0%)	Omitted			
**Highest educational achievement**						
No formal education	152 (45%)	41 (45.6%)	Ref		Ref	
Primary education	84 (24.9%)	20 (22.2%)	0.88 (0.49-1.60)	0.68		
Secondary education	14 (4.1%)	7 (7.8%)	1.85 (0.70-4.89)	0.21		
Post-secondary / Tertiary / Advanced education	88 (26%)	22 (24.4%)	0.96 (0.54-1.72)	0.89		
**Socio**-**economic class**						
Class 1	14 (4.1%)	2 (2.2%)	Ref			
Class 2	54 (16%)	13 (14.5%)	1.69 (0.34-8.35)	0.52		
Class 3	147 (43.5%)	48 (53.3%)	2.29 (0.50-10.40)	0.29		
Class 4	110 (32.5%)	25 (27.8%)	1.59 (0.34-7.45)	0.56		
Class 5	13 (3.9%)	2 (2.2%)	1.08 (0.13-8.79)	0.95		
**Current smoker**						
Yes	5 (1.5%)	2 (2.2%)	1.51 (0.29-7.93)	0.62		
No	333 (98.5%)	88 (97.8%)	Ref			
**Current alcohol user**						
Yes	28 (8.3%)	3 (3.3%)	0.38 (0.11-1.29)	0.12	0.28 (0.09-1.27)	0.09
No	310 (91.7%)	87 (96.7%)	Ref		Ref	
**Current herbal drug user**						
Yes	7 (2.1%)	3 (3.3%)	1.63 (0.41-6.44)	0.49		
No	331 (97.9%)	87 (96.7%)	Ref			

## Discussion

To our knowledge, this is one of the first longitudinal HIV immuno-virologic outcome and immuno-virologic discordance study done in large multi-center non-research public health facilities in Nigeria. Although favorable immuno-virologic outcomes could be achieved in this large ART program in which over half of the patients are on TDF-containing first-line, immuno-virologic discordance was observed in a third of the patients. Our findings provide important insights into specific predictors of immuno-virologic outcomes that are important for optimizing treatment strategies.

The patients were predominantly female reflecting the long standing feminization of the HIV epidemic in Nigeria [18], better health seeking behavior of women and possibly the linkage of treatment sites with the antenatal clinics and the prevention-of-mother-to-child HIV programs. Our study demonstrated that patients with higher baseline CD4 cell counts were statistically significantly more likely to have viral suppression at 12 months; a result in agreement with numerous other studies [19,20]. This well-known trend of late presentation and poorer male outcomes because of poor health seeking behavior of men [21] deserves renewed focus as male involvement in health program could improve decision-making not only for the men themselves but also for the women and children who live in male dominated societies. Men may need to be reached by new strategies for HIV voluntary counseling and testing focused on work and leisure sites frequently visited by men, flexible clinic hours for working men in addition to more aggressive mobilization campaigns targeted to men.

While many others had published on virologic outcomes results in Nigeria showing 62%-70% virologic suppression rates, they are mostly derived from a research setting [22-24]. The virologic suppression rate of 77% seen in this analysis is one of the first and the best seen in a large HIV treatment program from Nigeria and is consistent with a large review of 89 studies of antiretroviral programs in sub-Saharan Africa which showed a pooled virologic suppression rate of 76% at 12 months [25]. The robust immunologic response of > 50 cells / mm^3^ was seen in 77.4% of patients at 12 months with a median CD4 cell count increase of 139 cells / mm^3^ which is consistent with results obtained in other programs in similar settings [26,27]. Since more than half of the entire patient cohort were on a Tenofovir-containing regimen which is known for its superiority, convenient dosing, low toxicity and high potency [28-30], we were expecting a lower virologic failure rate than 23%. However, there were no statistically significant differences in virologic suppression by baseline drug regimen among this study cohort. These encouraging results confirm that favorable virologic and immunologic outcomes can be obtained from government-run busy large public health HIV treatment programs through technical assistance of PEPFAR implementing partners.

The finding of a strong association of virologic failure with anemia is noteworthy. Previous studies have associated anemia with poorer survival [31-33] and our findings suggest a 71% increased odds of virologic failure with anemia at baseline in addition to a 80% increased odds of virologic discordance. Apart from anemia caused by HIV itself and malnutrition, malaria and chronic helminthiasis are two most common causes of anemia in adults that respond to potent chemo-therapeutic interventions. Thus identifying and managing adults with anemia at baseline when HAART is being initiated as well as providing anti-helminthics can improve anemia and possibly retention and virologic outcome. A systematic review of 43 studies on the impact of hookworm infestation and anemia showed that treating non-pregnant adults with a combination of albendazole and praziquantel will increase hemoglobin level by a mean of 2.87 g/dl [34]. Based on the strong association of virologic failure and VL^-^/CD4^+^ discordance and anemia obtained in our study, chronic infections with anemia may be interfering with immuno-virologic recovery on HAART. We therefore recommend that a comprehensive treatment preparation and nutritional counseling program include anemia screening and management incorporating malaria and chronic helminthiasis treatment and provision of hematinics as a priority around the time of HAART initiation.

Younger age < 30 years was significantly associated with 79% increased odds of virologic failure and 50% increased odds of immunologic failure. A majority of studies including one previous evaluation of the ACTION program in Nigeria demonstrated an association between adherence and improved outcomes increasing with age [35-37]. Young people are more likely to be single, engage in high risk behaviors, lack social capital, financial ability and maturity [38-40] and thus should be prioritized in treatment preparation and adherence support programs.

Most studies including an extensive review of barriers to accessing HIV treatment and negative treatment outcomes suggest that longer distances from treatment sites are associated with poorer outcomes [37,41]. In our study, it appears that compared to those living less than 50 miles of the treatment site, those who lived 50–100 kilometers away had a 56% significantly decreased odds of virologic failure while those who lived the farthest (> 100 kilometers) had a 37% increased odds of virologic failure that was not significant. Since most Nigerians live close to the more than 300 HIV treatment sites in the country, it appears that adherent patients who travel > 50 kilometers to treatment centers make the personal choice to travel that long distance and are probably more motivated than patients who live close to the clinic. However, the farther the patients reside (> 100 kilometers) the greater the difficulty of handling the logistic and financial challenge of a far residential distance. A sub-analysis of our data confirmed that drug adherence by pharmacy refill visits was significantly best in the category of patients who live 50–100 kilometers from the treatment sites.

Even though socio-economic status have been linked to HIV treatment outcomes [42-44], our study surprisingly did not suggest an association between socio-economic or behavioral predictors to virologic failure. The most important impact of SES on HIV treatment is on the cost of the HIV drugs, laboratory work-up and the commodities. Under PEPFAR, HIV drugs and commodities (nutrition, bed-nets, and laboratory tests) are free in Nigeria; SES does not determine access to drugs and services possibly accounting for the lack of association. This may change in coming years as donors push for more country ownership of programs like PEPFAR with a greater share of costs supported by the public sectors. Educational achievement, particularly post-secondary education which has been previously associated with positive treatment outcomes [37,45] was associated with a 47% significantly reduced odds of virologic failure and 64% reduced odds of virologic discordance.

Immunologic failure normally occurs after virologic failure and can subsequently co-exist with each other. However, the associations of virologic and immunologic failure can differ since both conditions can also exist independently [46]. In our study, an increased odds of immunologic failure and immunologic discordance was associated with male gender. Male gender has been consistently associated with poor health seeking behaviors [47,48], lower baseline CD4 count levels and poor HIV treatment outcomes [37,49]. This underlines again the necessity of making many health care facilities male-friendly and encouraging male peer-support systems as well as investing in research and programs that adequately engage males and seek to positively influence the health-seeking behavior of men.

Immuno-virologic discordance has been defined and studied in multiple settings mainly in resource-rich countries and has been associated with co-infections (especially Cytomegalovirus infections), age, lower baseline CD4 cell count, poor adherence and high mortality [15,50-52]. In our study, the presence of other viral infections were not tested and only age and educational achievement was associated with immuno-discordance and no associations were seen with tuberculosis history and baseline CD4 counts as expected. Unfortunately, tuberculosis history was not consistently obtained and objective screening tests were not routinely performed as indicated.

In addition, our study results strongly suggest that viral load testing should be incorporated into routine patient care, both at baseline and at least every 6 months. Without viral load results, 16% (n=90) of our study population would be switched to a second line ART regimen on the basis of their CD4 count results even though they had sustained viral suppression. Conversely, 17% (n=94) of our study population who were eligible for second line ART regimen would have continued on first line therapy inappropriately, also based on their CD4 count result only. This confirms the results of many studies that CD4 cell count alone is a poor predictor of virologic and clinical failure and an unreliable guide to appropriate clinical management of HIV infected patients on antiretroviral therapy [53-55]. Viral load and CD4 measurements when done together will help in further identifying and characterizing immuno-virologic discordance which has been associated with increased risk of AIDS events and mortality [50,56]. In the Development of Antiretroviral Therapy in Africa (DART) trial [57] which was done in a clinical trials setting unlike the current study, clinical monitoring alone had a 31% increase in disease progression and mortality compared to clinical monitoring with routine laboratory monitoring implying benefit albeit small associated with prompt switching to second-line therapy.

Limitations of this study include less than ideal follow-up time (12 months), issues with measurement error, missing data and possible administrative error in recording variables common in busy government hospitals in resource-constrained settings. We use unimputed data and assumed all missing variables were missing completely at random (MCAR) and confirmed this assumption by doing multiple imputations (using sequential regression multivariate imputation (SRMI) method) which produced similar results. There were no baseline HIV RNA viral load results and all patients are expected to have high viral loads at baseline. However, we expect patients fully adherent on potent HAART regimen to be undetectable at 12 months after commencement of therapy. This study was conducted in non-pregnant adults and may not be generalizable to pregnant women and children. This study has some unique strengths including its clinic-based cohort design with validated immunologic and virologic results even though viral load testing were not routinely performed in this program. The selection of different levels of care and different locations serving different populations improved the generalizability of our findings. In addition, since Tenofovir is not a common first line drug in ART programs in Africa, these results could provide additional information on programs that predominantly use this drug in low and middle income countries.

## Conclusion

In summary, rapid HIV scale up has been hugely successful in Nigeria but challenges in patient retention and outcomes continue. With immuno-virologic outcomes known to worsen over time due to progressively poor adherence, pill fatigue and drug side effects, efforts should be intensified at baseline to identify patients who are likely to develop poor outcomes. Our study clearly shows that comprehensive baseline treatment preparation efforts needs to intensify adherence counseling, address anemia, male health seeking behavior, active case detection for opportunistic infections, affordable viral load testing, and regular CD4 cell count testing. In addition, younger patients (less than 30 years) and those with less than post-secondary education must receive priority attention in comprehensive treatment preparation and adherence support efforts. Future research efforts should be geared towards developing feasible and affordable routine viral load testing platforms and identifying whether operationalizing malaria and helminthiasis co-treatment will improve immuno-virologic outcomes. The role of sub-clinical or undiagnosed opportunistic infections in immuno-virologic discordance situations needs to be further evaluated.

## Endnote

^a^ This criterion was changed to CD4 count less than 350 cells / ml in 2010 in line with the WHO recommendation.

## Abbreviations

3TC: Lamivudine; ABC: Abacavir; ADH: Asokoro District Hospital, Asokoro, Abuja; AIDS: Acquired Immunodeficiency Syndrome; ARV: Antiretroviral; ART: Antiretroviral therapy; AUROC: Area Under Receiver Operating Curve; UATH: University of Abuja Teaching Hospital, Gwagwalada; AZT: Azidothymidine / Zidovudine; BMI: Body mass index; CD4: Cluster of differentiation 4 T-helper cells; D4T: Stavudine; EFV: Efavirenz; FTC: Emtricitabine; Global Fund: Global Fund to fight AIDS, TB and Malaria; HAART: Highly active antiretroviral therapy; HACART Study: HIV AIDS Care and Antiretroviral Therapy study; HIV: Human immune-deficiency virus; IHV: Institute of Human Virology, Baltimore, Maryland; IHVN: Institute of Human Virology Nigeria; LPV/r: Lopinavir boosted with ritonavir; LTFU: Loss to follow-up; MAP: Multi-country AIDS Program of the World Bank; M&E: Monitoring and Evaluation; MOHN: Ministry of Health, Nigeria; MTCT: Mother-to-child transmission of HIV; NHREC: Nigerian Health Research Ethics Committee; NNRTI: Non-nucleoside reverse transcriptase inhibitor; NRTI: Nucleoside reverse transcriptase inhibitor; NtRTI: Nucleotide reverse transcriptase inhibitor; NVP: Nevirapine; OI: Opportunistic Infections; PEPFAR: President Emergency Fund for AIDS Relief; PI: Protease inhibitor; PLWHA: Persons living with HIV AIDS; PMM: Patient monitoring and management forms; TB: Tuberculosis; TDF: Tenofovir disoproxil fumarate; UBTH: University of Benin Teaching Hospital, Benin; UNAIDS: Joint United Nations Agency for AIDS; VL: HIV viral load; WHO: World Health Organization

## Competing interests

The authors declare that they have no competing interests.

## Authors’ contributions

Conceived and designed the study: CA, WB, CB, MC. Study implementation: CA, AA, EE, HO, SA, OA. Data analysis: CA, MC. Contributed reagents / materials / analysis tools: CA, AA, WB, MC. Wrote paper: CA. Reviewed paper: CA, MC, CB, AA, ME, WB. Supervised patient care: EE, HO, SA, ME. Coordinated ACTION project: WB, PD, MC, AA, ME. All authors read and approved the final manuscript.

## Pre-publication history

The pre-publication history for this paper can be accessed here:

http://www.biomedcentral.com/1471-2334/13/113/prepub
